# A Hollow-Core Photonic-Crystal Fiber-Optic Gyroscope Based on a Parallel Double-Ring Resonator

**DOI:** 10.3390/s21248317

**Published:** 2021-12-13

**Authors:** Heliang Shen, Kan Chen, Kang Zou, Yijia Gong, Ran Bi, Xiaowu Shu

**Affiliations:** State Key Laboratory of Modern Optical Instrumentation, College of Optical Science and Engineering, Zhejiang University, Hangzhou 310027, China; 11930044@zju.edu.cn (H.S.); zoukang312@zju.edu.cn (K.Z.); 11830032@zju.edu.cn (Y.G.); biran@zju.edu.cn (R.B.); xwshu@zju.edu.cn (X.S.)

**Keywords:** fiber optic sensor, resonant fiber optic gyroscope, parallel double-ring resonator, hollow-core photonic-crystal fiber, Rayleigh backscattering noise, Kerr-effect-induced drift

## Abstract

A novel system structure of resonant fiber optical gyroscope using a parallel double hollow-core photonic crystal fiber ring resonator is proposed, which employs the double closed loop and reciprocal modulation–demodulation technique to solve the problem of the length mismatch between rings. This structure can suppress the residual amplitude modulation noise and laser frequency noise, essentially eliminating the influence of the Rayleigh backscattering noise and dramatically reduce the Kerr-effect-induced drift by three orders of magnitude. Thanks to its excellent noise suppression effect, the sensitivity of this novel system can approach the shot-noise-limited theoretical value of 8.94 × 10^−7^ rad/s assuming the length of the fiber ring resonator is 10 m.

## 1. Introduction

The resonant fiber optic gyroscope (RFOG) using a high-coherence light source can achieve high precision angular velocity measurement by detecting the resonance frequency difference caused by the Sagnac effect [1]. The RFOG has the potential to offer the same performance rotation sensing with a significantly shortened length compared with the commercial interferometric fiber-optic gyroscope (IFOG) [2]. This size advantage makes the RFOG very attractive for inertial navigation application. However, there are many parasitic effects, including Rayleigh backscattering noise [3], Kerr-effect-induced drift [4], temperature-driven polarization instability [5], residual amplitude modulation (RAM) noise [6], and laser frequency noise [7], deteriorating the performance of RFOG and making it far beyond the shot-noise-limited theoretical sensitivity.

Various methods have been reported for eliminating these parasitic effects. The Rayleigh backscattering noise can be reduced by phase modulators in both launching paths driven by different modulation frequencies, and using the phase modulators to suppress the carrier [3,8,9]. However, the use of separate modulation frequencies for clockwise (CW) and counterclockwise (CCW) waves, means that second harmonic distortion and RAM [6,9,10] will not be reciprocal to CW and CCW waves. Since these noises cause errors in the determination of the resonance center, they consequently translate to bias errors. The Kerr-effect-induced drift results from the light intensity difference between the CW and CCW waves circulating in the fiber ring resonator (FRR), which can be diminished by implementation of the light intensity feedback control technique [11,12,13] or adoption of spun fiber [14]. But research on these schemes have been restricted to complex structures that are not suitable for miniaturization. As for the drift caused by temperature-driven polarization instability, other groups have successfully reduced much of this drift by adding dual 90° polarization axis rotated splices [15] or adoption of a single-polarization resonator based on a micro-optical polarizing coupler [16]. These techniques require extremely precise assembly or difficult splices. Besides, splices or other polarization-dependent devices add undesirable loss and backscatter to the FRR. The emergence of hollow-core photonic-crystal fiber (HCPCF) offers a novel approach to reduce the Kerr-effect-induced drift [17,18] and polarization errors [19,20]. However, other experimental results indicated that the backscattering coefficient of some commercial HCPCFs are two orders of magnitude lager than the typical backscattering coefficient observed for SMF-28 fiber [21,22], which means the RFOG with a single HCPCF ring resonator may increase the Rayleigh backscattering noise.

From above, we find that these parasitic effects have been addressed by competing, or mutually exclusive countermeasures, and there is no countermeasure that can solve all these problems simultaneously. In this paper, we propose a novel system structure of RFOG using a parallel-double HCPCF ring resonator, collectively referred to as PDHC-RFOG. First, the Rayleigh backscattering noise in the PDHC-RFOG is analyzed in-depth. In addition, we establish a theoretical model to analyze the angular velocity drift induced by the Kerr effect in the PDHC-RFOG. Numerical simulations show that the Rayleigh backscatters in the PDHC-RFOG will not yield the demodulation error at the gyro output, and the PDHC-RFOG can reduce the Kerr-effect-induced drift by three orders of magnitude compared to the conventional RFOG using a single ring resonator. Moreover, the PDHC-RFOG is expected to suppress the temperature-driven polarization instability by a factor of 10 [20]. Applying the double closed loop [23] and reciprocal modulation–demodulation technique [10] to the PDHC-RFOG, it is encouraging that other parasitic performance barriers in the conventional RFOG, such RAM noise and laser frequency noise, can also be dramatically reduced.

## 2. The PDHC-RFOG System

**Figure 1:**
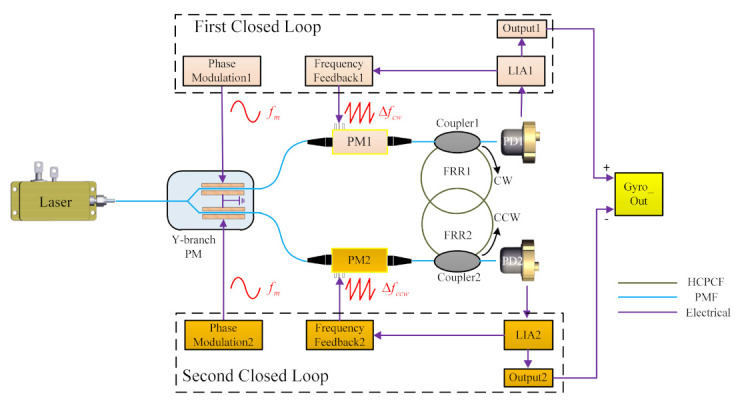
Schematic diagram of the PDHC-RFOG system.

A schematic diagram of the PDHC-RFOG employing the double closed loop [23] and reciprocal modulation–demodulation technique is depicted in Figure 1. The light emitted by the high-coherence laser is split into two equal-power beams (CW waves, CCW waves) by the LiNbO_3_ Y-branch phase modulator (Y-PM), and modulated with the same modulation frequency *f_m_*. The phase modulator 1 (PM1) is driven by a serrodyne wave for frequency shifting and PM2 is used for the same purpose [24]. Then the CW and CCW waves enter two HCPCF ring resonators through a 2 × 2 coupler, where fiber ring resonator 1 (FRR1) transmits CW waves, while ring resonator 2 (FRR2) transmits the CCW waves. A series of micro-optical lenses can be employed in the PDHC-RFOG as the 2 × 2 coupler, thus avoiding the polarization crosstalk in the resonator input coupler for conventional polarization-maintaining fiber [16,19]. Therefore, the light waves that originally propagated in the same FRR are changed to propagate separately in two independent HCPCF ring resonators without affecting each other. The CW and CCW waves from two HCPCF resonators are detected by photodetectors, PD1 and PD2, respectively. The output of PD1 is fed back through lock-in amplifier 1 (LIA1) and Frequency Feedback 1 to lock the laser frequency on the CW resonant frequency, which constitutes the first closed loop of the PDHC-RFOG. The output of PD2 is fed back through lock-in amplifier 2 (LIA2) and Frequency Feedback 2 to lock the laser frequency on the CCW resonant frequency, which constitutes the second closed loop of the PDHC-RFOG. Frequency Feedback 1 generates a serrodyne wave with an equivalent frequency shift of Δ*f_cw_*. Similarly, Frequency Feedback 2 generates a serrodyne wave with an equivalent frequency shift of Δ*f_ccw_*. Finally, the difference between two demodulation outputs gives the output of the rotation rate.

Taking the CCW waves as an example, the output intensity of the FRR2 normalized by the input intensity can be derived as:

(1) $$H_{ccw}\;=\;e^{-\alpha_{c}}\left[1\;-\;\rho \frac{{\left(1\;-\;Q\right)}^{2}}{{\left(1\;-\;Q\right)}^{2}\;+\;4Qsin^{2}(\omega \tau /2)}\right],$$

where

$$\left\{\begin{array}{l} \omega \;=\;2\pi f_{0}\\ \rho \;=\;1\;-\;\frac{{\left[T\;-\;(TQ+R)\right]}^{2}}{e^{-\alpha_{c}}{\left(1-Q\right)}^{2}}\\ T\;=\;e^{-\alpha_{c}/2}C_{bar}\\ R\;=\;C_{cross}^{2}e^{-\alpha_{c}}e^{-\alpha_{f}L/2}\\ Q\;=\;C_{bar}e^{-\alpha_{c}/2}e^{-\alpha_{f}L/2} \end{array}\right.;$$

where *f_0_* is the laser frequency; *C_bar_* and *C_cross_* are the coupling coefficients of the throughout arm and cross arm of the coupler, respectively; *α_f_* and *α_c_* are the additional loss of the fiber ring and coupler, respectively; *L* is the length of the FRR; *τ = n_0_L/c* is the transit time of the FRR, where *n_0_* is the refractive index of the fiber, and *c* is the speed of light in a vacuum.

The shot noise limited sensitivity is an import parameter of the RFOG, which can be expressed as [2,25]:

(2) $$\Delta \Omega \;\approx \;\frac{\sqrt{2}\lambda_{0}c}{LDF}\sqrt{\frac{hf_{0}}{\eta t_{i}I_{0}}}\frac{\sqrt{H_{max}}}{H_{max}\;-\;H_{min}},$$

where *λ*_0_ is the wavelength of the laser source, *D* is the diameter of the ring resonator; *F* is the finesse of the ring resonator; *η* is the photodetector quantum efficiency; *t_i_* is the integration time; *I*_0_ is the input optical power corresponding to the input electric field *E*_0_; *h* is Planck’s constant; and *H*_max_ and *H*_min_ represent the maximum and minimum of the resonance curve, respectively. Therefore, based on the Equations (1) and (2), and the parameters in Table 1, the shot-noise-limited theoretical sensitivity of the PDHC-RFOG is calculated to be 8.94 × 10^−7^ rad/s.

It is important to note that the FRR1 and the FRR2 should be the same type of HCPCF, and the placement of the ring resonator, ring diameter and initial length must be consistent as far as possible. However, it is difficult to have exactly the same length for both rings in practical applications. Therefore, we adopt the double closed-loop scheme to solve the inherent nonreciprocity problem of the PDHC-RFOG. Assuming that the length of the two rings is 10 m and the length mismatch between rings is controlled to within the order of one centimeter, thus the difference in the free spectral range between rings is calculated to be about 29.97 kHz. For the serrodyne modulation technique [24], only a serrodyne wave with a period of 33.367 μs is needed to enable the resonance of the light waves in the two rings, which can be realized theoretically.

Furthermore, the PDHC-RFOG using a double ring resonator structure allows both CW and CCW waves modulate by Y-PM with the same frequency, since the Rayleigh backscattering noise is naturally eliminated. In addition, this reciprocal modulation–demodulation technique makes the two counter-propagating light waves have the same RAM noise and laser frequency noise. Hence, the influence of these noises is eventually eliminated by differential output.

## 3. Suppression of the Rayleigh Backscattering Noise by the PDHC-RFOG

The Rayleigh backscattering noise limits the accuracy of the conventional RFOG. Although it has been proposed that the Rayleigh backscattering noise may be reduced by using HCPCFs since most of the mode travels through air instead of silica, but there were some measurements confirming that the backscattering coefficient for some commercial HCPCFs are still too high [21,22] due to dimensional fluctuations in the manufacturing process. Hence, the Rayleigh backscattering noise increases for conventional RFOGs with a single HCPCF ring resonator.

**Figure 2:**
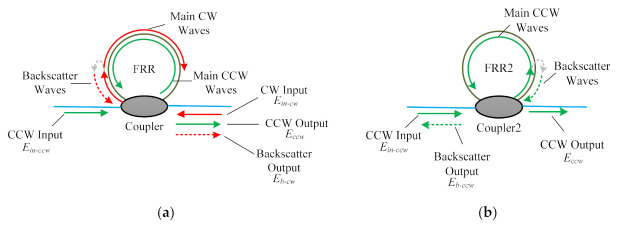
A simplified model for analyzing the Rayleigh backscattering noise in the CCW direction of the RFOG: with (**a**) a single HCPCF ring resonator; with (**b**) the parallel-double HCPCF ring resonator.

The PDHC-RFOG we proposed can essentially eliminate the influence of the Rayleigh backscatters. We analyzed the Rayleigh backscatters in the CCW direction of the conventional RFOG with a single-ring resonator and the PDHC-RFOG, respectively. Figure 2a shows the Rayleigh backscattering noise in the conventional RFOG, as the backscattered waves from CW waves encounter and interfere with CCW waves, the bias errors result. In addition, CW backscattered waves also cause errors from their own light intensity falling on the wrong photodetector.

The Equation (1) is applicable to both the conventional RFOG and the PDHC-RFOG. Moreover, as shown in the Figure 2a, the backscattered electric field of CW waves is given by

(3) $$E_{b-cw}\;=\;\frac{E_{0}C_{cross}^{2}e^{-\alpha_{c}}e^{-\alpha_{f}L}\sqrt{\alpha_{BS}}e^{i\left(\omega t\;-\;2\beta L\;-\;\theta \right)}}{{\left(1\;-\;Qe^{-i\omega \tau }\right)}^{2}},$$

where *α_BS_* is the Raleigh backscattering coefficient of fiber ring and *θ* is the phase difference between incident light waves and backscattered light waves.

Thus, in the conventional RFOG, the total electric field output is obtained as the sum of the Rayleigh backscattering, *E_b-cw_*, and the CCW waves, *E_ccw_*, which travel in the same direction as the backscatter, *E_b-cw_*. Then, the total intensity output, *I_out-single_*, is derived as

(4) $$I_{out-single}=\langle \left(E_{ccw}\;+\;E_{b-cw}\right)\;\cdot \;{\left(E_{ccw}\;+\;E_{b-cw}\right)}^{*}\rangle \;=\;I_{ccw}\;+\;I_{b-cw}\;+\;I_{i},$$

where the first term in Equation (4) is the CCW waves intensity given by Equation (1), the second term is the interference intensity between CCW waves and backscattered waves, and the last term is the backscattered waves intensity.

In contrast, in the PDHC-RFOG, the total electric field output only contains the CCW waves, *E_ccw_*, because the CW waves propagate in another HCPCF ring resonator, and there are no backscatters traveling in the same direction as CCW waves. Then, the total intensity output, *I_out-double_*, is given by

(5) $$I_{out-double}\;=\;\langle E_{ccw}\;\cdot \;E_{ccw}{}^{*}\rangle \;=\;I_{ccw}.$$

**Figure 3:**
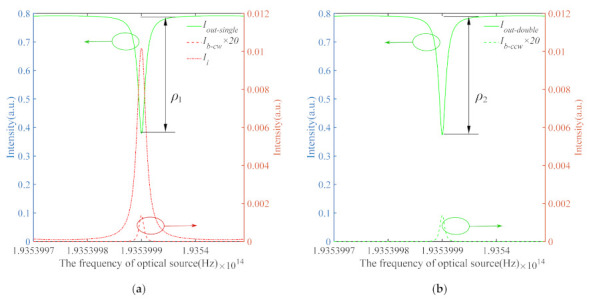
Simulated results of the Rayleigh backscattering noise in the CCW direction of the RFOG: with (**a**) a single HCPCF ring resonator; with (**b**) the parallel-double HCPCF ring resonator.

The simulation parameters are listed in Table 1. From the static simulation result in Figure 3a, it is clear that *I_b-__cw_* (red dash line) and *I_i_* (red dash-dotted line) have similar resonance characteristics to the CCW output, *I_out-single_* (green line). Therefore, the CCW output, *I_out-single_*, under the influence of the intensity term, *I_b-cw_*, and the interference term, *I_i_*, result in (1) reduction of the signal-to-noise ratio and (2) change of the resonance depth, *ρ*_1_. Even worse, the gyroscope output will fluctuate randomly due to the phase term, *θ*, not being constant in practical applications [26]. In contrast, Figure 3b shows the CCW output in the PDHC-RFOG, the two counter-propagating waves, *I_out-double_* (green line) and *I_b-ccw_* (green dash line), will not affect each other. Thus, only the CCW waves fall on the photodetector. Comparing Figure 3a and b, the resonance depth of the PDHC-RFOG *ρ*_2_ is 1.14% above *ρ*_1_.

## 4. Suppression of the Kerr-Effect-Induced Drift by the PDHC-RFOG

**Figure 4:**
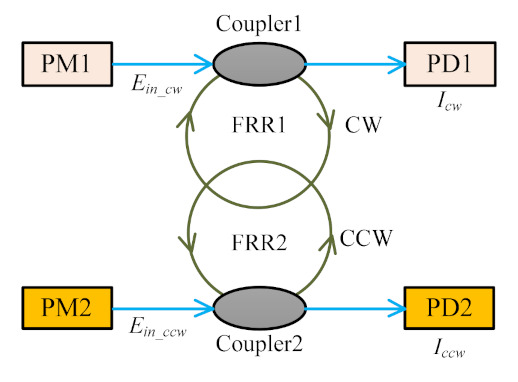
CW and CCW waves in the parallel-double HCPCF ring resonator.

The Kerr effect is one of the non-reciprocal optical effects that seriously deteriorates the performance of the RFOG. In the conventional RFOG, coherent optical propagation causes a large optical power density in the resonator owing to the small fiber diameter, as well as a significant change in the refractive index due to self-phase modulation (SPM) and cross-phase modulation (XPM) [4,27]. As shown in Figure 4, when the power of the CW waves and CCW waves in the FRR is unbalanced, a small non-reciprocal phase difference appears, which is superimposed on the non-reciprocal Sagnac phase difference causing a pseudo rotation drift signal, and eventually deteriorating the detection accuracy of the RFOG system.

The PDHC-RFOG we proposed is not affected by the XPM effect, but only by the SPM effect because the counter-propagating light waves are propagating in two separate HCPCF rings. Therefore, the perturbation of propagation constants *β_kcw_* and *β_kccw_* caused by the Kerr effect into two counter-propagating light waves is only related to its own light intensities *I_cw_* and *I_ccw_*:

(6) $$\begin{array}{l} \beta_{kcw}\;=\;\frac{2\omega Zn_{2}}{cA_{eff}}(I_{cw})\\ \beta_{kccw}\;=\;\frac{2\omega Zn_{2}}{cA_{eff}}(I_{ccw}) \end{array}$$

where *n*_2_ is the nonlinear refractive index coefficient caused by the Kerr effect in the fiber; *A_eff_* is effective area of the propagation mode in the fiber; *Z* is the impedance of the fiber; *u_cw_* and *u_ccw_* are the power-splitting coefficients of two counter-propagating waves divided by the Y-PM. The input electric field *E_in_cw_* and *E_in_ccw_* with finite temporal coherence and phase modulation [4] are expressed by Equation (7)

(7) $$\begin{array}{l} E_{in\_cw}\;=\;\sqrt{u_{cw}}\;\cdot \;E_{0}e^{j\omega t}e^{j\phi (t)}e^{jM_{cw}sin(\omega_{cw}t)}\\ E_{in\_ccw}\;=\;\sqrt{u_{ccw}}\;\cdot \;E_{0}e^{j\omega t}e^{j\phi (t)}e^{jM_{ccw}sin(\omega_{ccw}t)} \end{array},$$

where the phase fluctuation *ϕ*(*t*) represents the important parameter of optical source coherence; *ω_cw_* and *ω_ccw_* represent the angular frequency of sinusoidal phase modulation for CW and CCW waves respectively; *M_cw_* and *M_ccw_* represent the modulation index for CW and CCW waves respectively. Based on the theory of light wave field superposition, the intensities of two counter-propagating light waves *I_cw_* and *I_ccw_* are given by Equation (7) as

(8) $$\begin{array}{l} I_{cw}\;=\;A^{2}I_{0}\frac{1}{1\;-\;Q^{2}}\;\cdot \;\frac{1\;-\;Q_{0}^{2}}{1\;+\;Q_{0}^{2}\;-\;2Q_{0}cos(\varphi_{cw})}\\ I_{ccw}\;=\;A^{2}I_{0}\frac{1}{1\;-\;Q^{2}}\;\cdot \;\frac{1\;-\;Q_{0}^{2}}{1\;+\;Q_{0}^{2}\;-\;2Q_{0}cos(\varphi_{ccw})} \end{array},$$

where *A* is the transmission coefficient of light propagating in FRR; *φ_cw_* and *φ_ccw_* are the phase shifts in the CW and CCW directions caused by the Kerr effect and phase modulation. The operating points for two light waves are always fixed at the resonance. Thus, considering the resonance behavior of Equation (8)

(9) $$\varphi_{cw}\;=\;\varphi_{ccw}\;=\;2m\pi$$

is obtained. Where *m* is an integer. Therefore, combining with Equation (6), the angular velocity drift Ω_bias_ induced by the Kerr effect is derived as

(10) $$\Omega_{\mathrm{bias}}\;=\;\frac{c\lambda_{0}\Lambda }{2\pi LD}\;\cdot \;\left(\frac{u_{cw}}{1\;+\;\frac{1}{2}Q_{0}{\left[M_{cw}\omega_{cw}\tau /(1\;-\;Q_{0})\right]}^{2}}\;-\;\frac{u_{ccw}}{1\;+\;\frac{1}{2}Q_{0}{\left[M_{ccw}\omega_{ccw}\tau /(1\;-\;Q_{0})\right]}^{2}}\right),$$

where

$$\left\{\begin{array}{l} L\;=\;\frac{2\pi Zn_{2}}{\lambda_{0}A_{eff}}\;\cdot \;\frac{C_{cross}^{2}}{1\;-\;Q^{2}}\;\cdot \;\frac{1\;+\;Q_{0}}{\left(1\;-\;Q_{0}\right)}\;\cdot \;\frac{1\;-\;e^{-a_{f}L}}{a_{f}}\;\cdot \;e^{-a_{c}}I_{0}\\ Q_{0}\;=\;Q\;\cdot \;e^{-2\pi \Delta ft} \end{array}\right.$$

and Δ*f* is the linewidth of the light source. In addition, since we employ the same sinusoidal modulation scheme for both light waves, Equation (10) can be transformed into

(11) $$\Omega_{\mathrm{bias}}\;=\;\frac{c\lambda_{0}\Lambda }{2\pi LD}\;\cdot \;\left(\frac{u_{cw}\;-\;u_{ccw}}{1\;+\;\frac{1}{2}Q_{0}{\left[M_{cw}\omega_{cw}\tau /(1\;-\;Q_{0})\right]}^{2}}\right).$$

**Figure 5:**
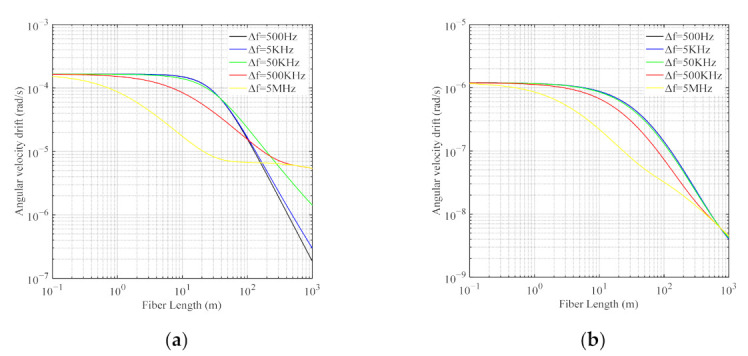
Angular velocity drift Ω_bias_ induced by the Kerr effect as a function of the ring resonator length *L* in the case of different light source linewidths. (**a**) Drift of the conventional RFOG with a sing SMF ring resonator. (**b**) Drift of the PDHC-RFOG.

With numerical evaluation, a plot of Ω_bias_ is shown in Figure 5 as a function of the ring resonator length *L*. Figure 5a shows that for the conventional RFOG with a single single-mode fiber (SMF) ring resonator [28] and Figure 5b for the PDHC-RFOG, respectively. The difference between the power-splitting coefficients |*u_cw_*-*u_ccw_*| is 0.01%, and other parameters are listed in Table 1 and Table 2.

Figure 5a illustrates that under the condition of constant linewidth of the light source, the drift Ω_bias_ induced by the Kerr effect decreases significantly as the ring resonator length *L* increases, while the system with a wider linewidth of the light source is less affected by the Kerr effect and can achieve lower drift Ω_bias_ with shorter fiber lengths. This is attributed to the change of the intensity in the FRR according to it fineness, which is closely dependent on its length and the light source linewidth. Hence, the Kerr effect noise has become the key performance barrier in the development of the RFOGs that require a shorter FRR length and narrower linewidth light source. Fortunately, this crucial problem can be settled by the PDHC-RFOG system. As shown in Figure 5b, the drift Ω_bias_ induced by the Kerr effect in the PDHC-RFOG is generally lower than that shown in Figure 5a.

**Figure 6:**
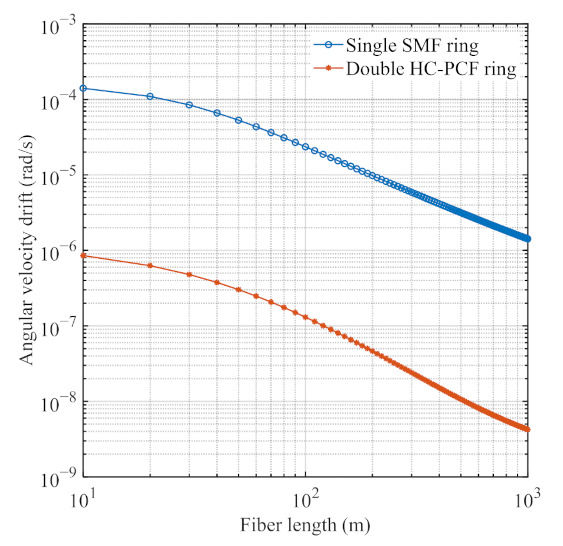
Comparison of angular velocity drift Ω_bias_ induced by Kerr effect when the light source has a fixed linewidth of 50 KHz: the blue line is the drift of the conventional RFOG with a sing SMF ring resonator, and the orange line is the drift of the PDHC-RFOG.

Figure 6 compares the drift Ω_bias_ induced by the Kerr effect in a single SMF ring resonator and double HCPCF ring resonator, when the light source has a fixed linewidth of 50 kHz. The figure shows that the drift Ω_bias_ of the PDHC-RFOG is better than the conventional RFOG with a single SMF ring resonator. The reason for this conclusion is that (1), compared to the SMF, the HCPCF fiber has a smaller nonlinear refractive index coefficient, *n*_2_, and (2) the structure of the parallel-double ring resonator allows the CW and CCW light waves to propagate in the separate ring resonator, further reducing the optical power density in the ring resonator, decreasing the difference in optical intensity due to transmission, coupling, and environmental factors. As a result, the drift Ω_bias_ of the PDHC-RFOG is reduced by about three orders of magnitude compared to the conventional RFOG with a sing SMF ring resonator. The drift Ω_bias_ of the PDHC-RFOG is 8.54 × 10^−7^ rad/s when *L* is 10 m, indicating that the Kerr-effect-induced drift is smaller than the shot-noise-limited theoretical sensitivity.

## 5. Conclusions

For reducing many of the parasitic noises limiting the bias performance of the conventional RFOG with a single ring resonator, we propose a novel system structure of RFOG using a parallel-double HCPCF ring resonator, namely, the PDHC-RFOG, which can effectively solve various problems caused by the parasitic noises. The PDHC-RFOG can essentially eliminate the influence of the Rayleigh backscattering noise and theoretically reduce the Kerr-effect-induced drift by three orders of magnitude. In addition, we adopt the double closed loop and reciprocal modulation–demodulation technique, which not only overcomes the problem caused by the length mismatch between rings but also suppresses the second harmonic distortion induced by RAM noise and laser frequency noise. The sensitivity of the PDHC-RFOG can approach the shot-noise-limited theoretical value. Based on the preceding analysis, it is evident that the PDHC-RFOG can achieve high stability and high precision angular velocity measurement.

## Figures and Tables

**Table 1:** Parameters for the PDHC-RFOG.

Parameter	PDHC-RFOG
*λ*	1550 nm
*L*	10 m
*D*	0.1 m
*n_0_*	1
*α*_BS_	2.5 × 10^−5^
*C*_bar_	$\sqrt{0.95}$
*C_cross_*	$\sqrt{0.05}$
*α_c_*	1 dB
*α_f_*	20 dB/km *
*I_0_*	1 mW
*η*	0.85
*t_i_*	1 s

* Value provided by NKT Photonics.

**Table 2:** Parameters for the RFOG with a single SMF ring resonator and the PDHC-RFOG.

Parameter	SMF-RFOG	PDHC-RFOG
*n_0_*	1.45	1
*f_m_*	50 kHz	50 kHz
*M*	2.405	2.405
*α_c_*	0.5 dB	1 dB
*α_f_*	0.2 dB/km **	20 dB/km *
*Z*	259.81 **	380.53 *
*n_2_*	2.6 × 10^−20^ m^2^/W **	2.89 × 10^−22^ m^2^/W *
*A_eff_*	8.5 × 10^−11^ m^2^ **	6.3 × 10^−11^ m^2^ *

* Values provided by NKT Photonics. ** Values provided by Corning Incorporated.

## References

[1] Lefèvre H. (1993). The Fiber-Optic Gyroscope.

[2] Meyer R.E., Ezekiel S., Stowe D.W., Tekippe V.J. (1983). Passive Fiber-Optic Ring Resonator for Rotation Sensing. Opt. Lett..

[3] Iwatsuki K., Hotate K., Higashiguchi M. (1984). Effect of Rayleigh Backscattering in an Optical Passive Ring-Resonator Gyro. Appl. Opt..

[4] Iwatsuki K., Hotate K., Higashiguchi M. (1986). Kerr Effect in an Optical Passive Ring-Resonator Gyro. J. Light. Technol..

[5] Watsuki K., Hotate K., Higashiguchi M. (1986). Eigenstate of Polarization in a Fiber Ring Resonator and Its Effect in an Optical Passive Ring-Resonator Gyro. Appl. Opt..

[6] Zhang C., Pan Z., Zheng Y., An P., Tang J., Liu J. (2019). Suppression of Residual Intensity Modulation Noise in Resonator Integrated Optic Gyro. Opt. Commun..

[7] Ma H., Zhang J., Wang L., Jin Z. (2017). Development and Evaluation of Optical Passive Resonant Gyroscopes. J. Light. Technol..

[8] Zhang Y., Feng L., Li H., Jiao H., Liu N., Zhang C. (2020). Resonant Fiber Optic Gyroscope with Three-Frequency Differential Detection by Sideband Locking. Opt. Express.

[9] Strandjord L.K., Qiu T., Salit M., Narayanan C., Smiciklas M., Wu J., Sanders G.A. Improved Bias Performance in Resonator Fiber Optic Gyros Using a Novel Modulation Method for Error Suppression. Proceedings of the 26th International Conference on Optical Fiber Sensors.

[10] Li H., Lin Y., Liu L., Ma H., Jin Z. (2020). Signal Processing Improvement of Passive Resonant Fiber Optic Gyroscope Using a Reciprocal Modulation-Demodulation Technique. Opt. Express.

[11] Ma H., Li X., Zhang G., Jin Z. (2014). Reduction of Optical Kerr-Effect Induced Error in a Resonant Micro-Optic Gyro by Light-Intensity Feedback Technique. Appl. Opt..

[12] Yin S., Liu W., Xing E., Pan Z., Tao Y., Zhu J., Tang J., Liu J. (2020). Suppression of Laser Intensity Fluctuation in Resonator Optical Gyro by a Simple Light Intensity Feedback Technique. Opt. Eng..

[13] Gong Y., Bi R., Shen H., Zou K., Chen K., Shu X. (2021). Influence of Intensity Noise on the Resonant Fiber Optic Gyroscope and Restraining. Opt. Commun..

[14] Wang Z., Wang G., Gao W., Cheng Y. (2021). Suppression of Kerr-Effect Induced Error in Resonant Fiber Optic Gyro by a Resonator with Spun Fiber. Opt. Express.

[15] Wang X., He Z., Hotate K. (2010). Reduction of Polarization-Fluctuation Induced Drift in Resonator Fiber Optic Gyro by a Resonator with Twin 90° Polarization-Axis Rotated Splices. Opt. Express.

[16] Jiao H., Feng L., Liu N., Yang Z. (2018). Improvement of Long-Term Stability of Hollow-Core Photonic-Crystal Fiber Optic Gyro Based on Single-Polarization Resonator. Opt. Express.

[17] Terrel M.A., Digonnet M.J.F., Fan S. (2012). Resonant Fiber Optic Gyroscope Using an Air-Core Fiber. J. Light. Technol..

[18] Sanders G.A., Taranta A.A., Narayanan C., Numkam Fokoua E., Abokhamis Mousavi S., Strandjord L.K., Smiciklas M., Bradley T.D., Hayes J., Jasion G.T. (2021). Hollow-Core Resonator Fiber Optic Gyroscope Using Nodeless Anti-Resonant Fiber. Opt. Lett..

[19] Sanders G.A., Strandjord L.K., Qiu T. Hollow Core Fiber Optic Ring Resonator for Rotation Sensing. Proceedings of the Optical Fiber Sensors.

[20] Zhao X., Louveau J., Chamoun J., Digonnet M.J.F. (2014). Thermal Sensitivity of the Birefringence of Air-Core Fibers and Implications for the RFOG. J. Light. Technol..

[21] Kim H.K., Dangui V., Digonnet M., Kino G. Fiber-Optic Gyroscope Using an Air-Core Photonic-Bandgap Fiber. Proceedings of the 17th International Conference on Optical Fibre Sensors.

[22] Ravaille A., Feugnet G., Fsaifes I., Baz A., Debord B., Gerome F., Humbert G., Benabid F., Bretenaker F. (2017). In-Situ Measurement of Backscattering in Hollow-Core Fiber Based Resonant Cavities. IEEE Photonics J..

[23] Suo X., Yu H., Li J., Wu X. (2020). Transmissive Resonant Fiber-Optic Gyroscope Employing Kagome Hollow-Core Photonic Crystal Fiber Resonator. Opt. Lett..

[24] Jin Z., Yu X., Ma H. (2013). Closed-Loop Resonant Fiber Optic Gyro with an Improved Digital Serrodyne Modulation. Opt. Express.

[25] Ying D., Demokan M.S., Zhang X., Jin W. (2010). Sensitivity Analysis of a Fiber Ring Resonator Based on an Air-Core Photonic-Bandgap Fiber. Opt. Fiber Technol..

[26] Huilian M., Huiquiang B., Zhonghe J. (2010). Backscattering in a Silica Optical Waveguide Ring Resonator. Chin. J. Lasers.

[27] Agrawal G.P. (2013). Nonlinear Fiber Optics.

[28] Xiujuan Y., Yanbiao L., Min Z., Youlong Y., Dejie L. (2008). Kerr Effect in an Optical Passive Ring-Resonator Gyro Using a Hollow-Core Photonic Band-Gap Fiber. Chin. J. Lasers.

